# Designing an optimized theta-defensin peptide for HIV therapy using in-silico approaches

**DOI:** 10.1515/jib-2023-0053

**Published:** 2025-03-19

**Authors:** Zahra Mosalanejad, Seyed Nooreddin Faraji, Mohammad Reza Rahbar, Ahmad Gholami

**Affiliations:** NoushDaru Intelligent Pars Company, Biotechnology Incubator, Shiraz University of Medical Sciences, Shiraz, Iran.; Department of Pathology, School of Medicine, 48435 Shiraz University of Medical Sciences, Shiraz, Iran; Nush Darouye Hooshmand Pars Company, Biotechnology Incubator, 48435 Shiraz University of Medical Sciences, Shiraz, Iran; Pharmaceutical Sciences Research Center, 48435 Shiraz University of Medical Sciences, Shiraz, Iran; Department of Pharmaceutical Biotechnology, School of Pharmacy, 48435 Shiraz University of Medical Sciences, Shiraz, Iran; Biotechnology Research Center, Shiraz University of Medical Sciences, Shiraz, Iran

**Keywords:** HIV, theta defensin, glycoprotein 41 (gp41), molecular docking, peptide design

## Abstract

The glycoproteins 41 (gp41) of human immunodeficiency virus (HIV), located on the virus’s external surface, form six-helix bundles that facilitate viral entry into the host cell. Theta defensins, cyclic peptides, inhibit the formation of these bundles by binding to the GP41 CHR region. RC101, a synthetic analog of theta-defensin molecules, exhibits activity against various HIV subtypes. Molecular docking of the CHR and RC101 was done using MDockPeP and Hawdock server. The type of bonds and the essential amino acids in binding were identified using AlphaFold3, CHIMERA, RING, and CYTOSCAPE. Mutable amino acids within the peptide were determined using the CUPSAT and Duet. Thirty-two new peptides were designed, and their interaction with the CHR of the gp41 was analyzed. The physicochemical properties, toxicity, allergenicity, and antigenicity of peptides were also investigated. Most of the designed peptides exhibited higher binding affinities to the target compared to RC101; notably, peptides 1 and 4 had the highest binding affinity and demonstrated a greater percentage of interactions with critical amino acids of CHR. Peptides A and E displayed the best physiochemical properties among designed peptides. The designed peptides may present a new generation of anti-HIV drugs, which may reduce the likelihood of drug resistance.

## 1 Introduction

The human immunodeficiency virus (HIV) specifically targets certain critical immune system cells, particularly CD4^+^T cells. HIV continues to be a global health crisis, with 39 million individuals living with the virus in 2022 and 630,000 deaths attributed to AIDS that year [1]. HIV can be categorized into two subtypes: HIV-1 and 2, in which the former is known to be more infectious and virulent compared to the latter [2]. In Central Africa, HIV-1 Group M diversified into multiple subtypes in the twentieth century: A, B, C, D, F, G, H, J, K, and L [3].

One of the proteins on the surface of the HIV membrane is the glycoprotein 160 complex, which comprises glycoproteins 120 (gp120) linked to a glycoprotein 41 (gp41), forming a homotrimer structure. During virus entry, gp120 interacts with CD4 on the mature virion surface, promoting the accumulation of CD4 and chemokine receptors [4]. Subsequently, gp120 interacts with a chemokine receptor and fully dissociates from gp41, facilitating the viral membrane’s fusion to the host cell. This process involves gp41 dynamic conformational changes [5]. The N-terminal heptad repeat 1 (NHR) region of the gp41 molecule becomes exposed, While the C-terminal or heptad repeat 2 (CHR) is bent towards the grooves formed by the NHRs, resulting in the formation of a six-helix bundle (6HB). Finally, the viral membrane fuses with the cell membrane [6].

There are currently 25 medications available for AIDS management, categorized into six main classes. A standard anti – HIV regimen typically consists of three drugs [7], which patients must take for life. Despite ongoing treatment, the virus is constantly exposed to the drug without being entirely eradicated, leading to various mutations. Investigating approaches is needed due to drug resistance and side effects, and exploring new treatment strategies is essential [8].

Drugs that inhibit the binding of HIV to the host cell are promising and have shown good safety and efficacy, with minimal drug interactions and resistance. However, large-scale clinical trials are required to confirm these alternatives [9]. Enfuvirtide, a 36-amino acid peptide and a viral fusion inhibitor targeting gp41, was the first FDA-approved HIV-entry inhibitor [10]. It is derived from CHR and is effective against different subtypes of HIV-1 but does not work against HIV-2 [11].

Defensins are small cationic peptides classified into alpha, beta, and theta categories in mammals, with antimicrobial activity against various pathogens, including bacteria, fungi, protozoa, and viruses [12]. They target several stages of the virus life cycle through binding to viral or host proteins [13], [14]. Theta-defensin (TD) analogs have shown effectiveness against HIV strains resistant to enfuvirtide [15].TDs, cyclic peptides found in old-world monkeys, are encoded by a human gene but are not naturally expressed [14]. Synthetic peptides, called retrocyclins (RC), have been developed using human pseudogenes. Six TDs have been identified, including RC 1, 2, 3, and three types from rhesus monkeys [16]. TDs have a unique structure with two antiparallel β-strands stabilized by three disulfide bonds that form a cyclic cystine ladder [17]. These peptides exhibit high stability, low toxicity, and low hemolytic properties and are resistant to temperature changes, proteolysis, salt, and serum, with minimal immunogenicity in animal models. Compared to other defensins, TDs are almost insensitive to salt, divalent cations, and serum [18]. TDs block early stages of HIV entry by binding to carbohydrate-containing molecules like gp120 and CD4 and disrupting gp41 function, particularly by binding tightly to the CHR region, preventing the formation of the 6-helix bundle [19], [20]. Analogs of TDs such as DpVs and RC-valine have been demonstrated to have anti-HIV effects [21], [22]. Retrocyclin-101 (RC101), the most successful TD analog, was modified by replacing arginine at position 9 with lysine, enhancing its activity against multiple HIV-1 subtypes by binding to the CHR of gp41 [23], [24].RC101 exhibits strong antiviral activity with low cytotoxicity in various models and does not trigger inflammatory cytokines [25], [26]. Bioinformatics techniques allow efficient analysis and prediction of peptide properties with less cost and time [27], [28], [29]. This study used in-silico methods to design new peptides with higher affinity to CHR than RC101, investigating their physicochemical properties, pharmacokinetics, toxicity, and antigenicity.

## 2 Methods

### 2.1 Tetha-defensin peptide structure

The structure of human theta-defensin (TD) was retrieved from the RCSB Protein Data Bank (https://www.rcsb.org/) [30]. Of the two available structures (2ATG and 2LZI), 2LZI was selected based on the molprobity score chart (Figure S2) available at https://bmrb.io/data_library/summary/index.php?bmrbId=18757. The amino acid sequences of all available TDs were extracted from the RCSB, and structural alignment was performed using Clustal Omega (https://www.ebi.ac.uk/Tools/msa/clustalo/) [31]. The RC101 structure was designed by mutating 2LZI using Pymol software (version 2.5.2) (https://pymol.org//) [32]. Structure refinement was carried out with Galaxy Refine (https://galaxy.seoklab.org/) [33], and the Ramachandran plot of the models was analyzed at https://swift.cmbi.umcn.nl/servers/html/ramaplot.html to select the best-refined structure.

#### 2.1.1 Mutation analysis and designing new peptides

To identify mutable amino acids of RC101, Duet [34] (http://structure.bioc.cam.ac.uk/duet) and Cupsat (http://cupsat.tu-bs.de/) [35] were utilized. CUPSAT (Experimental Method Thermal, using amino acid-atom potentials and torsion angle distribution to assess the amino acid environment of the mutation site). Each amino acid is systematically mutated to 19 other possible amino acids, identifying those that enhance or maintain molecular stability or function post-mutation. Mutations that improve performance, stability or both were selected. (Intra-molecular bonds in RC101 were analyzed using the RING server (https://ring.com/) [36]. Finally**,** based on the results from Duet and Cupsat’s, as well as the conserved and essential amino acids of TDs and CHR, 32 new peptides were designed.

### 2.2 Glycoprotein structure of HIV

The gp160 structures of HIV were extracted from UniProt (https://www.uniprot.org/) [37], and the alignment of various HIV subtypes was performed using Clustal Omega. Conserved regions were identified during this process. The structure of gp41 was retrieved from the RCSB database, and 1AIK was selected among several structures. The NHR chain was removed (leaving the CHR chain) using Chimera software (version 1.15) (https://www.cgl.ucsf.edu/chimera/) [38]. The structure was then refined using Galaxy Refine and the Ramachandran plot analysis to select the best-refined structure. Hot Spot Wizard (https://loschmidt.chemi.muni.cz/hotspotwizard) [39] was used to identify regions where amino acids frequently changed during evolution.

### 2.3 Molecular docking analysis

The molecular docking of CHR with RC101 and designed peptides was conducted using MDockPeP (https://zougrouptoolkit.missouri.edu/mdockpep) [40] to compare their binding affinities. MDockPeP is an ab initio peptide and protein docking server that evaluates interactions without prior knowledge of binding sites. This server, validated by the PeptiDB database, ranks binding modes generated from various peptide conformations based on their binding energy scores (ITScorePeP), a scoring function based on the statistical potential created for protein-peptide bindings [41].

Peptide numbers 1 and 4 (Table 1) were selected due to their low ITScorePeP values (IT score < −250). The bond types and essential interactions between the selected peptides and CHR were analyzed using CYTOSCAPE and RING. Other servers were also used to investigate the connections of peptides 1 and 4 with CHR. Docking with CHR through MM/GBSA analysis was done at the HawDock server (http://cadd.zju.edu.cn/hawkdock) [42]. HawkDock server uses the global docking algorithm implemented in ATTRACT and the HawkRank scoring function, as well as the identification of the critical residues by MM/GBSA. MM/GBSA (Molecular mechanics with generalized Born and surface area solvation) is employed to predict the binding free energy and decompose the free energy contributions to the binding free energy of a protein-protein complex in per-residue.

**Table 1:** The name and sequence of the designed peptides and ITScorePep of their docking with CHR in MDOckpep. Regions highlighted in red are the mutated amino acids_._

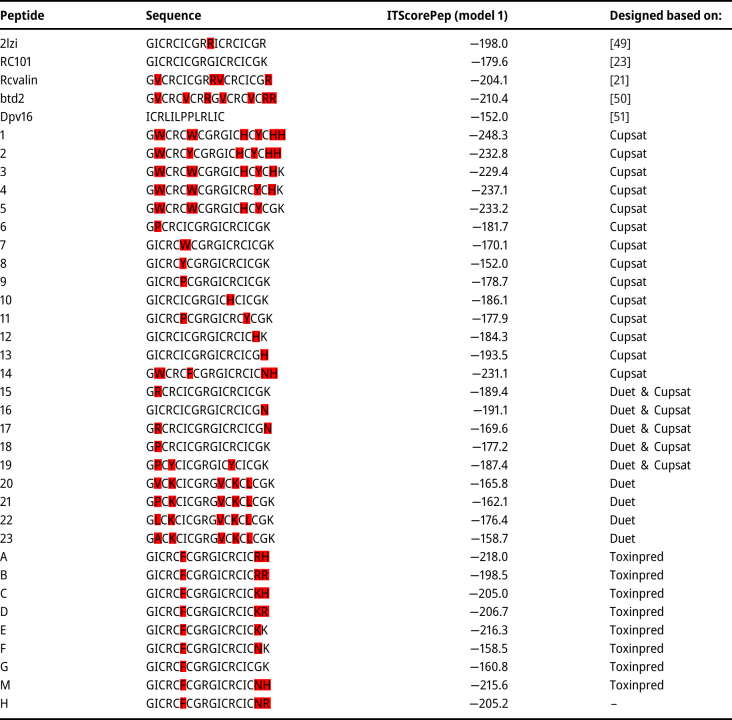

The 3D structure was created by Pymol and AlphaFold3. The sequences of peptides 1, 4, RC101, and 2LZI, each along with CHR, were given to Alphafold3 software. The final complex was given to the RING to check the interactions, and MM/GBSA analysis was performed.

#### 2.3.1 Interaction interface analysis

Interaction networks between amino acids of two molecules were identified using Cytoscape at https://cytoscape.org [43]; the RINanalyzer Cytoscape plugin was used to create a network of amino acid interactions by connecting UCSF Chimera to Cytoscape. Then, betweenness central analysis (BCA) was performed, which evaluates the number of times a node is passed by paths between all pairs of nodes, considering the shortest paths.

### 2.4 Properties of designed peptides

Antigenicity and allergenicity level of the peptides (ITScorePep less than −200) was checked from the Vaxijen (http://www.ddg-pharmfac.net/vaxijen/VaxiJen/VaxiJen_help.html) and Allertop (https://www.ddg-pharmfac.net/AllerTOP/index.html) [44], [45]. Allertop has a dataset of different allergenic and non-allergenic peptides and makes predictions by comparing the input peptides with them. The toxicity of peptides was predicted with toxinpred (http://crdd.osdd.net/raghava/toxinpred/). The dataset of this server contains 1805 toxic peptides with a length equal to or less than 35 amino acids [46]. Also, some peptides with less toxicity are designed based on this server. Using Protparam (https://web.expasy.org/protparam/) [47] and (Peptide property calculator) pepcalc (https://pepcalc.com/), some physicochemical and pharmacokinetic properties of the peptides were checked [48].

## 3 Results

### 3.1 Peptide structure and designing new peptides

Chain number 5 of 2lzi (NMR) extracted from RCSB (Figure S1) was selected in Chimera due to the lower MolProbity score (higher quality) (Figure S2). Results of alignment showed that ARG (numbers 4, 9, 13) are highly conserved (Figure S3). The structure of RC101 was obtained by mutating the 2lzi molecule with Pymol. The results of the DUET and CUPSAT servers are shown in Table S3.

Thirty-two peptides were designed (based on Duet and Cupsat’s results, conserved and essential amino acids of TDs and CHR, Tables S1–4 and Figures S3 and 4).

### 3.2 Glycoprotein of HIV

By gp41 alignment, conserved amino acids among HIV subtypes were identified (Figure S4).

The NHR chain was removed (the CHR chain remained). The CHR structure was refined by Galaxy refine, and the best model was selected by comparisons of the Ramachandran plot (Figure S5).

**Figure 1:**
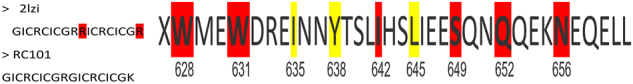
left: comparison sequence of 2LZI and RC101. Right: CHR, essential amino acids in binding CHR to NHR (yellow), conserved-essential amino acids (red).

Essential amino acids in the CHR-NHR connection were identified by investigating articles and are called essential amino acids (EAA). Amino acids number 628, 631, 642, 649,652, and 656 are essential and also conserved, which we are called them conserved-essential amino acids (CEAA) (Figure 1, Table S2). The amino acids of CHR that have undergone many mutations over time were identified by the Hot Spot Wizard (Table S4). The possibility of changing these amino acids is higher in the future. None of the amino acids predicted in the Hot Spot Wizard were among the CHR conserved or essential amino acids (except GLU 659, listed as essential amino acids in some articles).

### 3.3 Docking of RC101 with CHR

**Figure 2:**
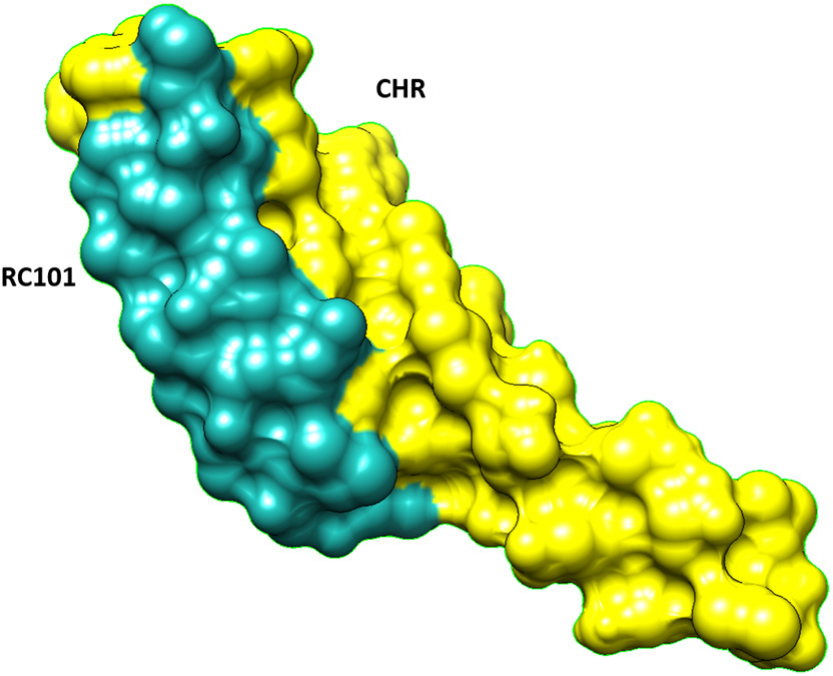
The first docking model (Mdockpep) of RC101 and CHR interaction.

RC101docked with CHR. The docking file of the first model (the highest affinity (among the top ten models) was downloaded. (ITPep score: −179) (Figure 2).

### 3.4 Docking of designed peptides with CHR

The designed peptides docked with CHR by MDockpep. The binding affinity to CHR of other TDs analogs in past studies, BT2, DPV16, and RC valine, was also checked. The ITScorePep of peptides 1, 2, 3, 4, 5, 4, and A, E, M, and H were below −200. (Compared to the rest of the peptides, they had a higher binding affinity to CHR and had more negative binding energy. Among these peptides, peptides 1 (−248.3) and 4 (−237.1) had the highest binding affinity. In contrast, ITScorePep RC101 was calculated as −179.6 (Table 1, Figure S6).

The 3D structure of the best-designed peptides (ITScorePep less than −200) was constructed with pymol, and re-docking was performed to obtain more accurate results. Peptides 1 and 4 still have the most affinity, and their ITScorePep became more negative in Docking (Table 2: Peptide 1, −254.3 and peptide 4, −251.6).

**Table 2:** Docking results of peptides and CHR, the 3D structure of peptides with ITScorePep less than −200 was made, and the docking results were compared with sequence input alone. Peptides 1 and 4 had better scores in both cases.

Best designed peptide	IT ScorePep (first model) with sequence	IT ScorePep (first model) with 3D structure	Hydrophobicity	Charge
2lzi	−214.9	−198.0	−0.29	5.00
RC101	−176.9	−179.6	−0.14	4.00
0	−227.6	−231.1	−0.18	3.50
1	−248.3	−254.3	−0.14	3.50
2	−232.8	−238.3	−0.16	3.50
3	−229.4	−245.1	−0.18	4
5	−228.6	−233.2	−0.25	4.50
4	−237.1	−251.6	−0.15	3.50
A	−218.0	−213.5	−0.22	4.50
E	−216.3	−208.0	−0.22	5
H	−205.2	−209.7	−0.23	4.00
M	−210.7	−215.6	−0.16	3.50

### 3.5 Centrality analysis of interaction networks

**Figure 3:**
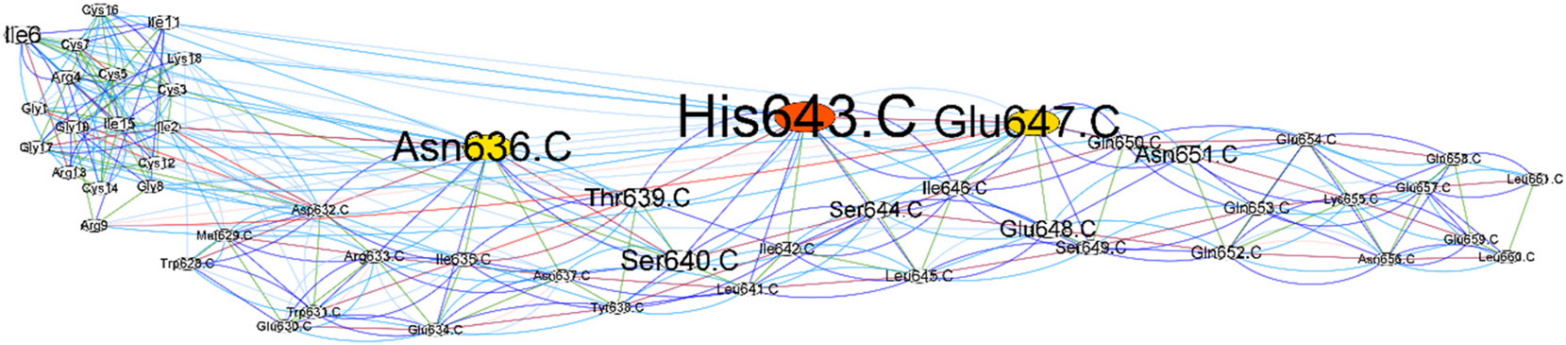
The network displayed by betweenness centrality analysis in Cytoscape software, the interaction of RC101-CHR, nodes represent amino acids, and lines represent identified interactions. Also, the color spectrum of *Z* score = 2 is shown in yellow to *Z* score = 4 in red. The higher amino acid *Z* score is displayed as a larger size. The node size is proportional to the *Z*-score.

**Figure 4:**
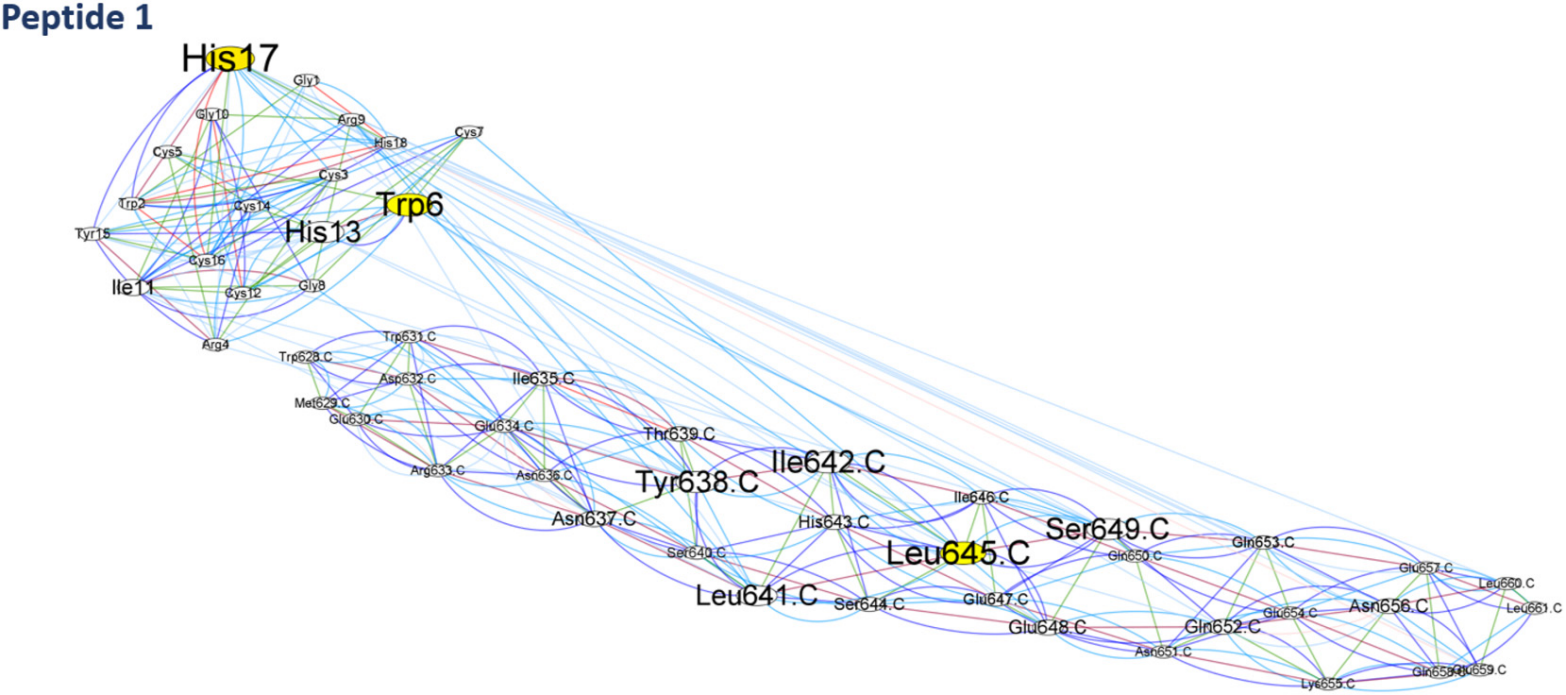
The network displayed by betweenness centrality analysis in Cytoscape software, peptide 1-CHR interaction, nodes represent amino acids, and lines represent identified interactions. Also, the color spectrum of *Z* score = 2 is shown in yellow to *Z* score = 4 in red. The higher amino acid *Z* score is displayed as a larger size.

**Figure 5:**
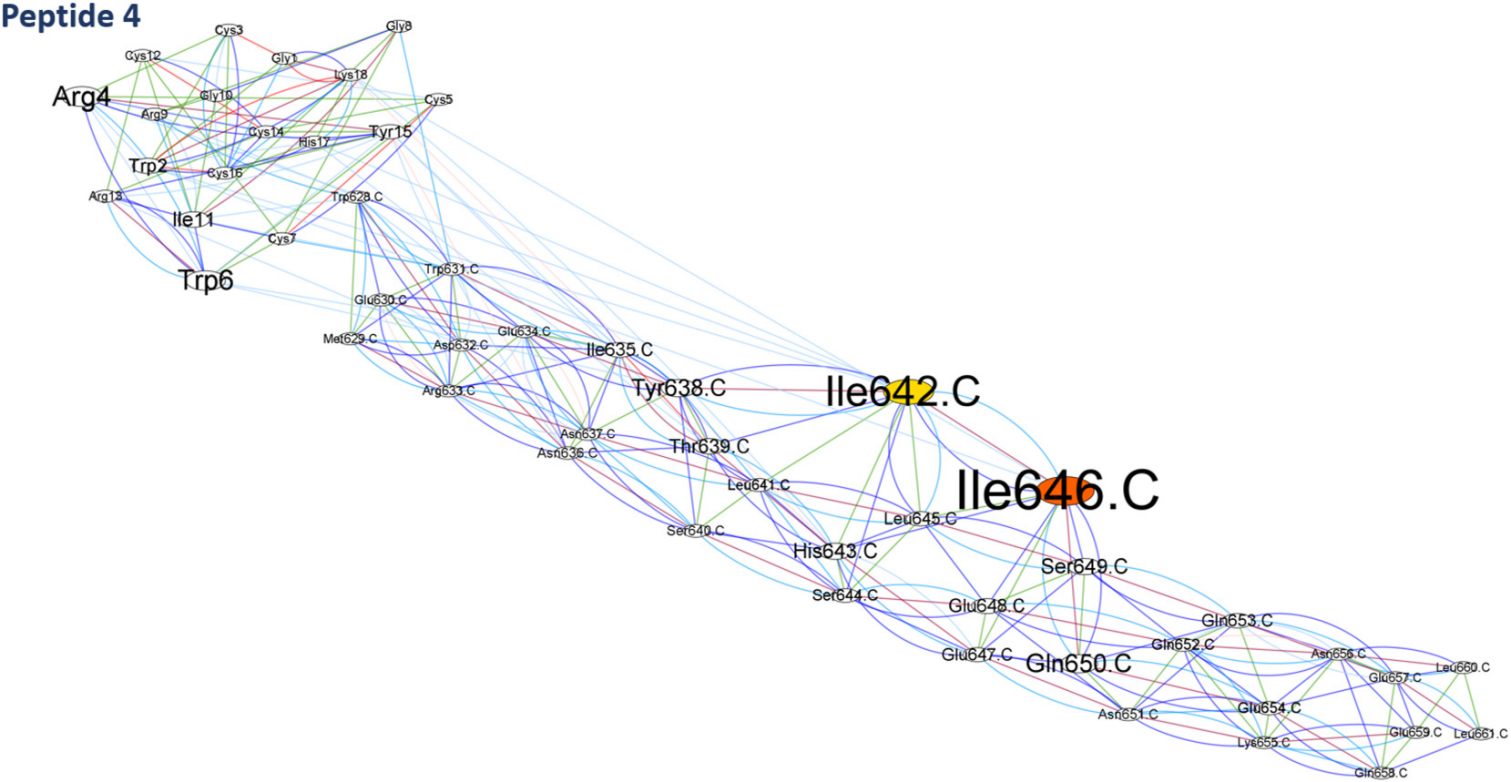
The network displayed by betweenness centrality analysis in Cytoscape software, the interaction of peptide 4-CHR, nodes represent amino acids, and lines represent identified interactions. Also, the color spectrum of *Z* score = 2 is shown in yellow to *Z* score = 4 in red. The higher amino acid *Z* score is displayed as a larger size.

BCA analysis (Betweenness Centrality Analysis) was performed using Cytoscape software (Figure 3). The amino acids that have *Z* score ≥ 2: for peptide 1 docking, Leu645 in CHR Trp6 His17 (Figure 4); for peptide 4, Ile 642,646 of CHR (Figure 5), and RC101, Asn636, His643, and GLU647 of the CHR have *Z* score ≥ 2 (Figure 3).

The amino acid) node (has a higher *Z* score in the network, which increases the average shortest path length by removing it from the network. (Usually, a score higher than or equal to 2 is significant), so it probably plays a more critical role in connecting structures [52].

### 3.6 CHR-peptides interaction interface

**Figure 6:**
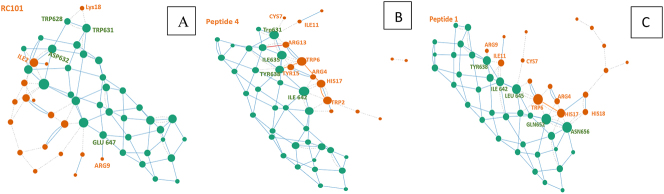
CHR-peptides interaction interface using RING. (A) Binding of peptides (orange) (A: RC101, B: peptide 4, C: peptide 1) to CHR (green), circles indicate amino acids. Dotted lines represent van der Waals bonds, and complete lines represent hydrogen bonds. In this figure, both intramolecular and intermolecular bonds are displayed.

The designed peptides 1 and 4 have established more bonds with CHR. Also, more of their bonds are established with CEAA of CHR. (57.9 in peptide 1, 46.6 in peptide 4 and 14.3 % in RC101) (Table 4, Figure 6).

Among the mutated amino acids of peptide 1, TRP6 and HIS17 established the most bonds with CHR. (4 van der Waals bonds) Moreover, in second place are H13 and 18 with one van der Waals bond. Among the mutated amino acids of peptide 4, TRP6 has two van der Waals bonds and one pi bond, ranking first in the number of bonds with CHR. The other three mutated amino acids establish two van der Waals bonds with CHR (Tables 3–5).

**Table 3:** In investigating the bond type of peptides with CHR, the CHR’s amino acids are written first, and in front of it, the name and number of the amino acids of the peptide with which form a bond are written. Essential CHR amino acids are highlighted in yellow, and essential-conserved amino acids are highlighted in red. If an amino acid of CHR has more than one bond with a peptide’s amino acid, it is indicated after the peptide number.

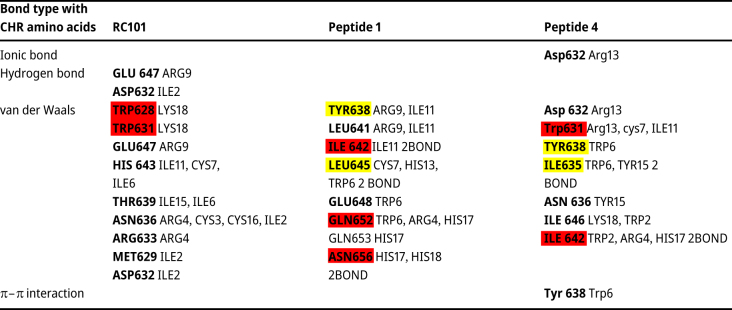

**Table 4:** Comparison of peptides 1 and 4 and RC101 regarding the number of bonds with CHR and the percentage of bonds with essential and essential-conserved amino acids.

Number of bonds with: peptide	CHR conserved and essential amino acids	CHR essential amino acids	Total number of bonds	The percentage of bonds with conserved-essential aa to the total bonds (van der Waals)
RC101	2 van der Waals	–	14 van der Waals 2 hydrogen bonds	14.3
Peptide 1	11 van der Waals	6 van der Waals	19 van der Waals	57.9
Peptide 4	7 van der Waals	van der Waals 4 1 *π*–*π* interaction	15 van der Waals 1 *π*–*π* interaction 1 ionic bond	46.6

**Table 5:** The number of bonds amino acid bonds of mutated peptides 1 and 4 with CHR.

Number of bonds Van der Waals bonds		W2	W6	H13	Y15	H17	H18		W2	W6	Y15	H17
With conserved and essential amino acids	**Peptide 1**	0	1	0	0	2	1	**Peptide 4**	1	0	0	2
With essential amino acids		0	2	1	0	0	0		0	2 + 1 *π*–*π*	2	0
Total Van der Waals bonds		0	4	1	0	3	1		2	3	3	2

### 3.7 Intramolecular bonds

The intramolecular hydrogen bonds of peptide 1, such as RC101, were 7, and peptide 4 had 6 hydrogen bonds. However, the intramolecular van der Waals bonds increased from 3 in RC101 to 8 and 6 in peptides 1 and 4, and peptide 1 also had a *π*–*π* bond (Table 6).

**Table 6:** Comparison of the number and type of intramolecular bonds of RC101 with peptides 1 and 4.

Peptides	Hydrogen bond	*π*–*π* interaction	Van der Waals
RC101	7	–	3
Peptide 1	7	1	8
Peptide 4	6	–	6

### 3.8 Final analysis of peptide 1, 4

Hawdock’s score of 2lZI is −2,565.2. The results of peptides 1, 4 and RC101 differed depending on the 3D structure building model (first and second column of Tables 7 and 8).

**Table 7:** The difference in predicting the binding affinity of peptide to CHR using different methods in building 3D structure of peptide (AlphaFold3, pymol) and scoring functions (MM/GBSA analysis, Hawdock score).

Peptides	Hawdock score (using pymol for peptide structure)	Hawdock score (using AlphaFold3 for peptide structure)	MM/GBSA analysis (using pymol for peptide structure and docked by Hawdock) (kcal/mol)	MM/GBSA analysis (using AlphaFold3 for peptide structure docked by Hawdock) (kcal/mol)	MM/GBSA analysis interaction prediction (using AlphaFold3) (kcal/mol)
2LZI	−2,565.27	−2,565.27	−46.81	−46.85	−37.45
RC101	−2,460.75	−2,619.78	−39.34	−41.05	−15.87
Peptide 1	−2,340.15	−2,423.18	−43.58	−46.66	−11.24
Peptide 4	−2,433.36	−2,731.34	−41.15	−39.17	−43.93

**Table 8:** Comparison of binding affinity of peptides to CHR using different methods.

Method	Comparison binding affinity to CHR
Hawdock score (using pymol for peptide structure)	2LZI > RC101 > Peptide 4 > Peptide 1
Hawdock score (using AlphaFold3 for peptide structure)	**Peptide 4 > RC101 > 2LZI >** Peptide 1
MM/GBSA analysis (using pymol for peptide structure and docked by Hawdock)	2LZI > Peptide 1 > Peptide 4 > RC101
M/GBSA analysis (using AlphaFold3 for peptide structure docked by Hawdock)	2LZI > Peptide 1 > RC101 > Peptide 4
MM/GBSA analysis interaction prediction (using AlphaFold3)	**Peptide 4 > 2lZI > RC101 >** Peptide 1

#### 3.8.1 Analysis of interaction

**Figure 7:**
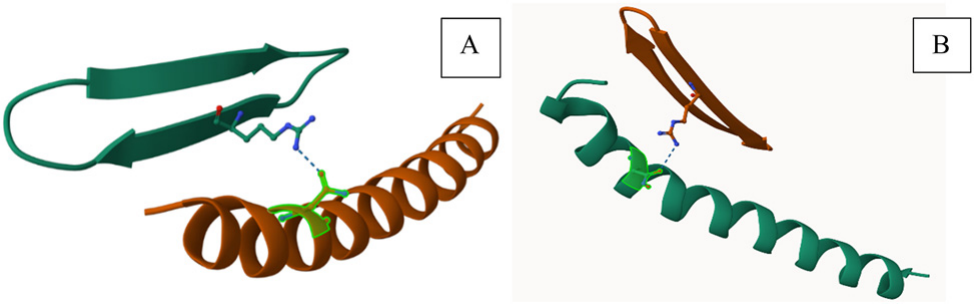
Peptides’ hydrogen bonds with CHR using RING. (A) Peptide 1, hydrogen bond with CHR (ARG4 peptide 1: ASN636 CHR). (B) Peptide 4, hydrogen bond with CHR (ARG13 peptide: ASN636 CHR).

The RING results are written in Table 9 for further investigation of interaction. Hydrogen bonds were observed in peptides 1 and 4 binding to CHR (Figure 7).

**Table 9:** The bonds between peptide and CHR using Alphafold3, RING (essential amino acids in binding CHR to NHR [yellow], conserved-essential amino acids [red]).

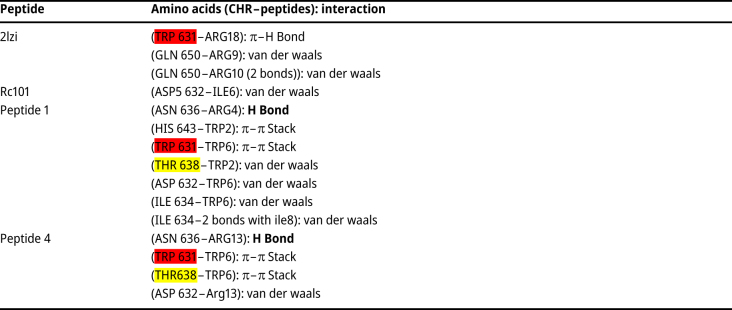

**Table 10:** MM/GBSA analysis (kcal/mol), binding affinity of peptides to CHR (conserved-essential amino acids [red]).

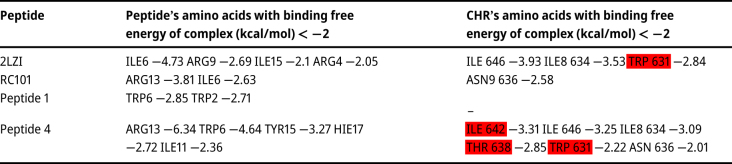

The results of the MM/GBSA analysis are listed in Table 10.

**Table 11:** Investigating the physicochemical properties of the peptide with toxin pred.

Peptide	Hydrophobicity	Charge
2lzi	−0.29	5.00
RC101	−0.14	4.00
0	−0.18	3.50
1	−0.14	3.50
2	−0.16	3.50
3	−0.18	4
4	−0.25	4.50
5	−0.15	3.50
A	−0.22	4.50
E	−0.22	5
H	−0.23	4.00
M	−0.16	3.50

### 3.9 Antigenicity, toxicity, and physicochemical properties of selected peptides

Unlike RC101 and 2lzi, designed peptides did not have bacterial antigenicity. Peptides 1, 2, 3, and M had parasite antigenicity. 2Lzi 1, 2, 4, and 2 were predicted to be non-allergenic. Among the selected peptides 1 and 4, peptide 4 had less antigenicity. All peptides except those 0, 1, 2, 3, 4, and 5 were considered non-toxic. Hydrophobicity values vary from −2 to +2 for most proteins; proteins with a positive score are more hydrophobicity. All peptides have negative values and are hydrophilic (Table 11 and S5).

Half-lives of all peptides are estimated at 30 h. All peptides predicted stable except peptides 1, 2, 3, and 4 [53]. Almost all peptides predicted to be stable had a high aliphatic index (except peptide 5). A high aliphatic index indicates a protein is stable over a wide temperature range. Peptides 1–5 are relatively hydrophilic. All designed peptides have a positive charge (Table S5).

Peptides A and E showed the best physiochemical properties among designed peptides. (Most positive charge, stable, non-toxic, good water solubility, and minimum antigenicity).

## 4 Discussion

In this study, 32 new peptides were designed based on the RC101 peptide, which is the most successful analog of TDs in the treatment of HIV. Designed peptides differ from one to 6 amino acids from RC101. The best peptides (The most affinity to CHR) were selected. Moreover, some of their essential physicochemical and pharmacokinetic properties were investigated. We tried to target the CEAA of CHR. Also, amino acids that probably mutated in the future were identified using the Hot Spot wizard server, which should be paid less attention to for peptide design. By targeting CEAA amino acids, peptides can act against different subtypes, so the viruses can hardly develop resistance against them. In a study, a mutation in the NHR region caused relative resistance to the RC101, But the mutation in CHR decreased the fitness and function of the virus [21]. Docking of Rc101 with gp41 shows that it binds more strongly to CHR. Most of the mutations that can render HIV-1 resistant to RC-101 also have deleterious effects on the ability of HIV-1 itself to initiate infection [24].

The genetic difference between different subtypes of HIV is significant. For example, the virus’s envelope protein has an average of 25 % amino acid difference [54]. For a drug to cover most of the world’s subtypes, it must be effective against subtypes A, B, and C [55]. Although there are differences in HIV subtypes, based on the alignment results of CHR, there are common conserved amino acids, some of which are important in binding CHR to NHR. For selecting the gp41 structure, 1AIk was selected [56] due to its proper resolution, and it is subtype B, one of the increasing subtypes [57]. Also, In a study, a structure that bound a trimer with a deleted ligand like (1AIK with deletion of NHR) was introduced as a suitable structure for docking [58]. Considering the different conformations of gp41 during virus binding to the host cell, the part of the molecule used in docking does not change much [59].

Computational analysis and in-silico investigations to identify novel epitope designs for achieving new drugs have shown promising results in infectious diseases [60], [61]. For designing new peptides, cysteines and their position remain constant [49] because the peptide’s cyclic structure is preserved during evolution. The preserved hydrophobic, hydrophilic pattern remained [62], So in regions where hydrophilic amino acid was always placed during evolution, the hydrophilic amino acid was replaced. RC-111 had the same amino acid sequence as RC-1. However, its amino acids were reversed, and the *in vitro* study increased the infection of cells! This shows that the topology and polarity of TDs affect their type of effect against HIV [63].

In designed peptides still have **Glycine (Gly)** that has hydrogen in the side chain instead of carbon. It makes the structure more flexible. Gly plays an essential role in hairpin structures and is conserved in TDs because of the formation of beta turns [64]. Arginine (Arg) and Gly are presented in both turns [65], [66].

Positive amino acids are preserved or replaced with positive-charged amino acids [67] because the CHR has a negative charge; therefore, a positive charge in peptides is necessary [24]. Passage of HIV-1 under selective pressure by RC101 resulted in a 5-10-fold reduction in viral susceptibility to RC101. Each mutation replaced an electronegative or electroneutral amino acid with a positively charged amino acid, consistent with the peptide binding to the anionic parts of gp41 [68]. **Arg** is often involved in salt bridges, interacting with a negatively charged amino acid (such as aspartate). Arg of RC-1 plays an essential role in carbohydrate binding. Therefore, the mutated peptides still have Arg.

Most of these amino acids in peptides 1 and 4 were replaced with isoleucine (Ile), which has branched hydrocarbon side chains. Ile probably causes reversible self-association of TDs [69]. Furthermore, it may have a negative effect on binding peptides to the target. Peptides 1 and 4 still have some Ile. Tryptophan (Trp) can inhibit the binding of CHR to NHR By binding to the hydrophobic pocket of gp41 [70]. The hydrophobic pocket is located in the C-terminal part of NHR and connects to the pocket-binding domain (PBD) region of the six-helix bundle. Which includes hydrophobic amino acids Trp and Ile (Trp 628, Trp 631, and Ile 635) [71] due to the presence of Trp in the PBD region, probably the presence of aromatic amino acids in TDs can help improve properties. The hydrophobic pocket is a suitable area for binding inhibitors of this protein [72]. Also, studies have shown that the aromatic amino acids of lectins can play a role in binding to the hydrophobic parts of monosaccharides [73]. Tyrosine (Tyr) and Trp are aromatic amino acids in peptides 1 and 4.

In addition, both peptides 1 and 4 also contain the amino acid histidine (His); His has a pKa close to physiological pH, so it is relatively easy to move protons in the side chain (i.e., change the side chain from neutral to positive charge) and is also an ideal amino acid for functional protein centers.

RC101, BCA analysis in Cytoscape, Asn636, His643, and GLU647 of CHR had Z scores above 2, which are not essential or conserved CHR amino acids. In addition, Asn636 was predicted in the Hot Spot Wizard server as a highly mutated amino acid.

In peptide 1, Trp6 His17 amino acid and Leu645 in CHR had a Z score above 2, which are mutated amino acids in the peptide. Moreover, Leu645 is one of the essential amino acids of CHR.

In peptide 4, Ile 642,646 of the CHR had a Z score above 2. Ile 642 is one of the CEAA. This can explain the high binding affinity of peptides.

RC101 makes two hydrogen bonds with CHR. (GLU 647 ARG9, ASP632 ILE2) One of the amino acids is Arg 9, which is not mutated in peptides 1 and 4, so there is a possibility of this connection in them as well.

To form an ix-helix bundle, it is necessary to create a salt bridge between Asp632 of the C-terminal part of the heptad repeat and Lys574 in the N-terminal part [74]. In RC-101, Lys is placed instead of arginine amino acid, strengthening the effect [64]. Also, Ile 2 formed a hydrogen bond in docking and van der Waals bonds with Asp 632. This amino acid is mutated to Trp in peptides 1 and 4. Trp also can establish a hydrogen bond through the nitrogen of the indole group. Trp is non-polar, the same as Ile. However, it is the largest size amino acid. Lys 18 forms a van der Waals bond with two CEAAs, Trp628 and Trp631.

The mutated amino acids of peptide 1, Trp6, and His17 and 18 with two CEAA (ASN656, GLN 652) establish a van der Waals bond.

CHR’s amino acid asparagine 656 (Asn-145) should interact with valine (Val-38) on NHR. (In fact, Asn-145 together with Leu-149 Glu-146 create a hydrophobic site) [75]. Arg13 in peptide 4 makes ionic and van der Waals bonds with Asp632. The peptide has mutated amino acids, Trp 2 and His17, establishing van der Waals bonds with CEAA (Ile642). Also, peptide 4 makes a *π*–*π* bond with EAA (Tyr 638) through Trp 6**.**

Human TDs (retracycline) were chosen to have minimal antigenicity in humans, and the RC101 analog properties were derived from human TDs. Regarding antigenicity, peptide 4 is lower than RC101 antigen; parasitic antigenicity was predicted for peptide 1. However, peptides 1 and 4 had no bacterial antigenicity, unlike RC101. Peptides designed based on toxinpred were not toxic. However, peptides 1 and 4 were predicted to be toxic, probably due to their higher binding affinity. Therefore, it should minimize toxicity through targeted drug formulation and delivery, although constrained peptides exhibit fewer drug side effects [76].

The charge of all designed peptides is positive; as a result, they can better bind to CHR, which has a negative charge [77]. The isoelectric pH of the designed peptides ranged from 8.35 to 9.30. Because the peptide has no charge at this pH, dissolution, and function (binding to CHR) decreases. The pH of the human body in a healthy state is between 7.35 and 7.45 (on average, 7.40) [78]. Therefore, these peptides will have a positive charge in the environment of the human body.

Peptides 1 and 4 were predicted to be hydrophilic based on the GRAVY index and toxinpred. However, according to the Pepcalc site, they did not have a suitable water solubility that can be solved with appropriate formulation [79].

The stability of designed peptides decreased, but due to the cyclic structure of TDs and three disulfide bonds, these peptides are still considered stable molecules and have the appropriate properties of a constraint peptide [80]. Also, in investigating the peptides’ intramolecular bonds the van der Waals number inside Peptides 1 and 4 is more than RC101 (Table 6).

TDs bind to multiple components of the target cell membrane (such as glycoproteins, glycolipids, and glycosaminoglycans) and can create high concentrations in that region [20]. Making a fusion pore may require 1 to 7 Env spikes for entry stoichiometry; most HIV strains depend on 2–3 Env spikes [81]. In previous studies, it has been shown that in gp41 inhibitory peptides, for proper binding affinity, the peptide should target at least two binding pockets [82].

In the turn of TDs, smaller molecules can be attached [83] that bind to CD4+ or gp120 to produce an anti-HIV drug with several mechanisms. Also, the concentration of the peptide near the virus is increased.

Peptide inhibitors have been developed to inhibit six-helix bundle formation, peptides derived from NHR or CHR that bind to gp41 fusion intermediates [84], [85].

For the design of newer peptides, these things can be considered: resistance to proteolytic degradation, optimization of solubility properties, and higher binding affinity to the target [75].

In an *in vitro* study, several advantages for using TDs to inhibit HIV have been mentioned, including the need for a short time (10 min) to be effective and in low concentrations (0.2–1 g/ml) [63], and Constrained peptides such as TDs are good choices for drug development [80]. Also, the small size of these peptides makes them more accessible to their target [86].

The difference result was observed in predicting the binding affinity of peptide to CHR using different methods in building the 3D structure of peptide (AlphaFold3, pymol) and scoring functions methods (MM/GBSA analysis, Hawdock score, and IT pepscore).

According to an article, MDockpep introduces the best peptide and protein docking for peptides with a length of 16–20 amino acids [87], but after completing this study, the server does not accept peptides with more than 15 amino acids [40].

In the analysis of the bonds’ type predicted by MDockpep and Alphafold3, which was performed by RING, the interaction of peptides 1 and 4 with EAA (THR 638) and CEAA (TRp631) was observed, and peptides make hydrogen bonds with CHR (Figure 7). In MM/GBSA analysis, mutated amino acids of peptides 1 and 4 (TRPp 2, TRP6) were also observed among the amino acids with more negative binding energy. This can be consistent with the design logic.

This study is limited to silico studies. We also obtained different results by using different docking programs and analyses. According to the results of all docking programs, it seems that between peptides 1 and 4, peptide 4 is more suitable for future studies. And it probably shows more binding affinity to CHR *in vitro*.

## 5 Conclusions

Among the peptides derived from the RC101, peptides 1 and 4 had the highest binding affinity to the CHR. The mutated amino acids of peptides 1 and 4 had more bonds with CHR than other amino acids. Also, they contributed more to establishing important bonds (bonding with conserved or essential amino acids of CHR).

These two designed peptides can be proposed as an option in the treatment regimen of people with HIV, especially in people who have become resistant to other drugs or experience severe adverse effects in treatment. These two peptides are effective against various HIV_1 Subtypes, and less resistance will be developed against them; laboratory studies should confirm these results.

## Supplementary Material

Supplementary Material Details
