# Supplementary material for: Designing an optimized theta-defensin peptide for HIV therapy using in-silico approaches
Source: J Integr Bioinform. 2025 Mar 19;22(1):20230053. doi: 10.1515/jib-2023-0053 (PMC12327201; doi:10.1515/jib-2023-0053)
Supplement: Supplementary file 1 — Supplementary Material Details [file j_jib-2023-0053_suppl_001.docx]

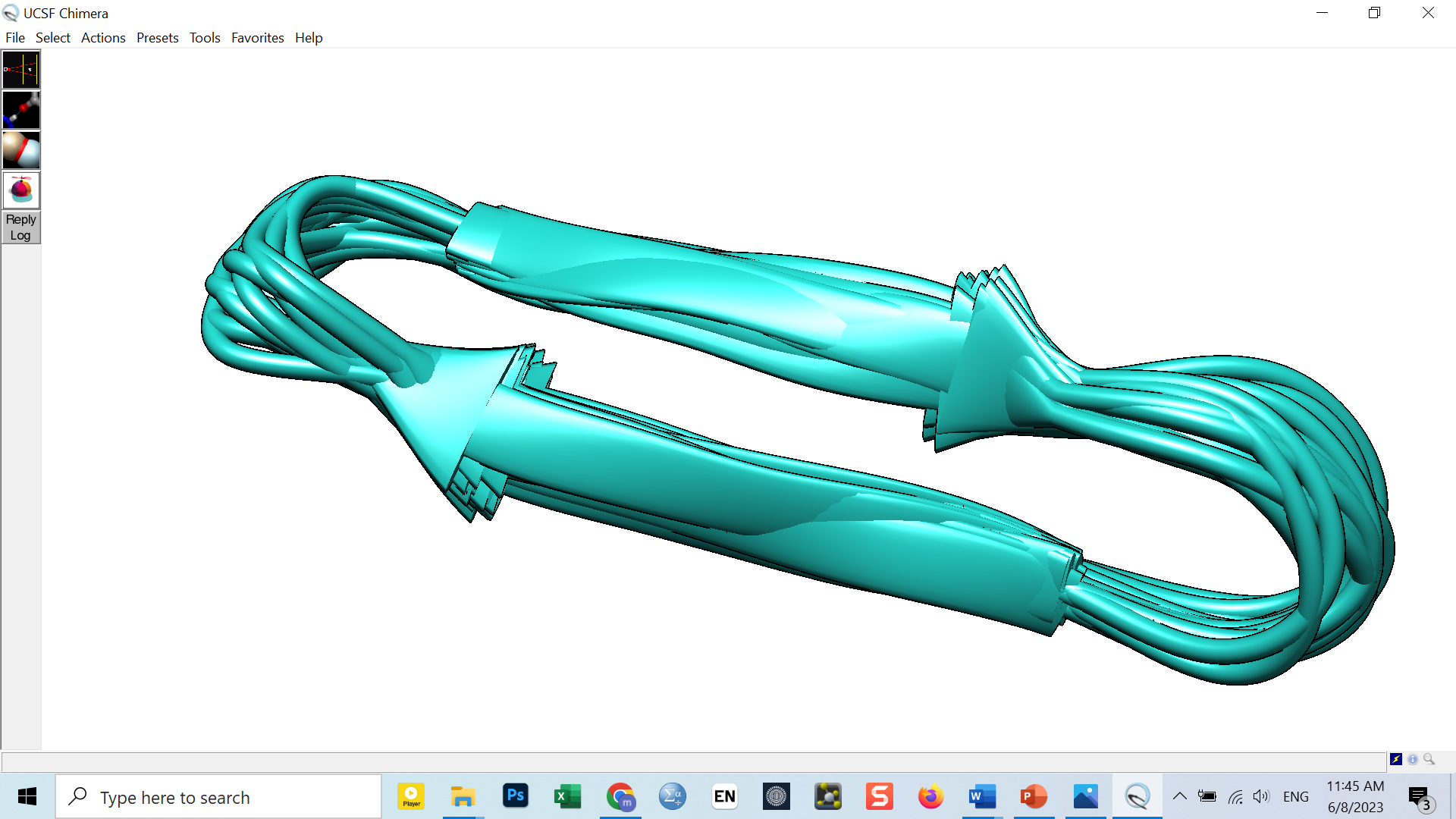


**Figure S1.** The three-dimensional structure of the selected human Theta Defensin (2lzi) ribbon displayed in the chimera software.


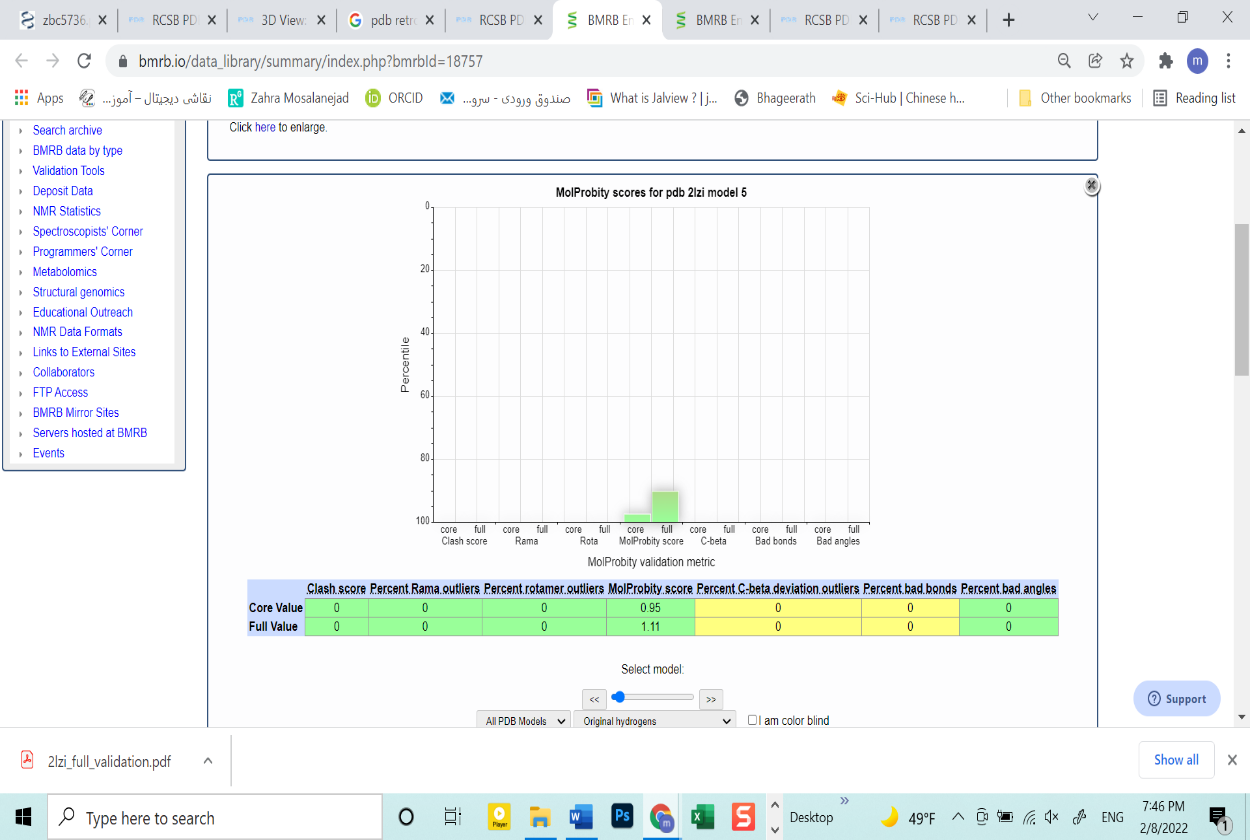


**Figure S2.** MolProbity diagram of chain number 5 in the NMR structure of Theta Defensin (2lzi), which has the lowest MolProbity among the 20 chains in the structure.


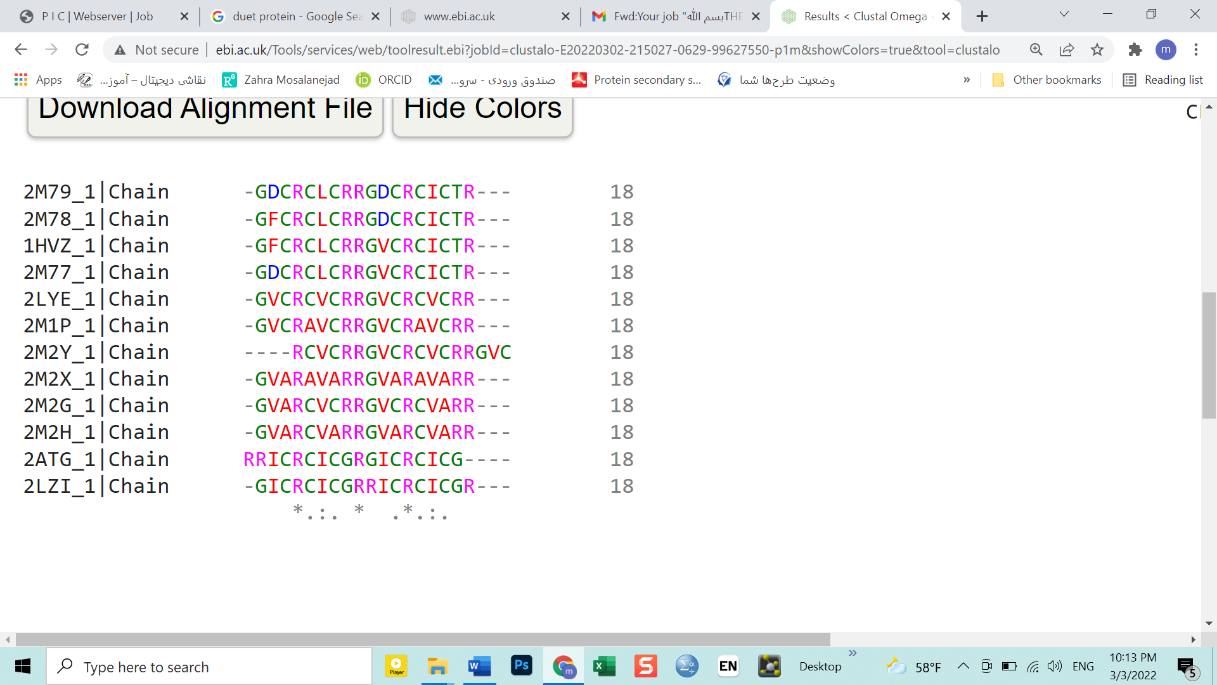


**Figure S3.** Alignment of theta defensins in RSCB, using Clustal omega, there are two structures of human Theta Defensin (2ATG, 2lZI).


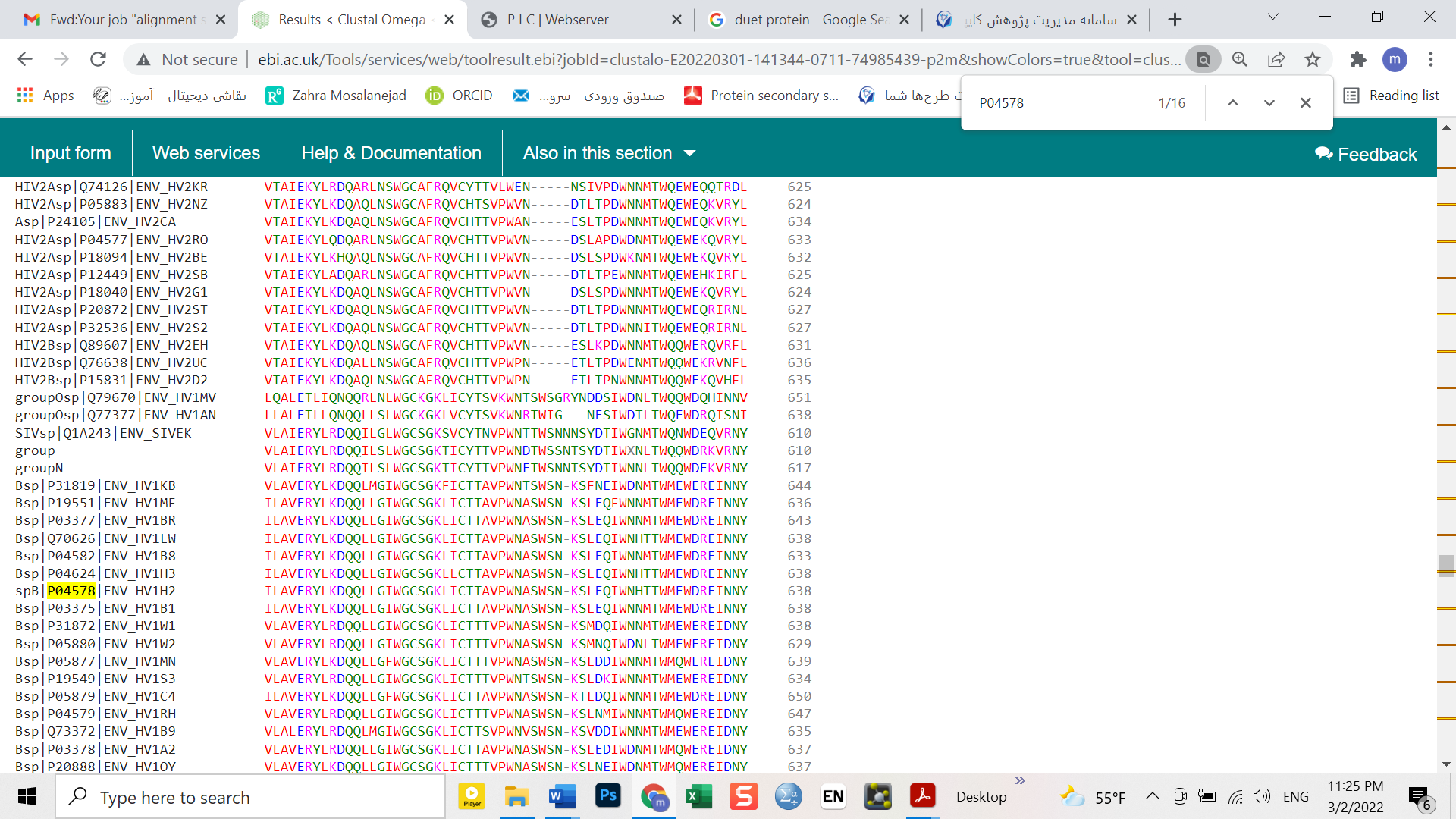

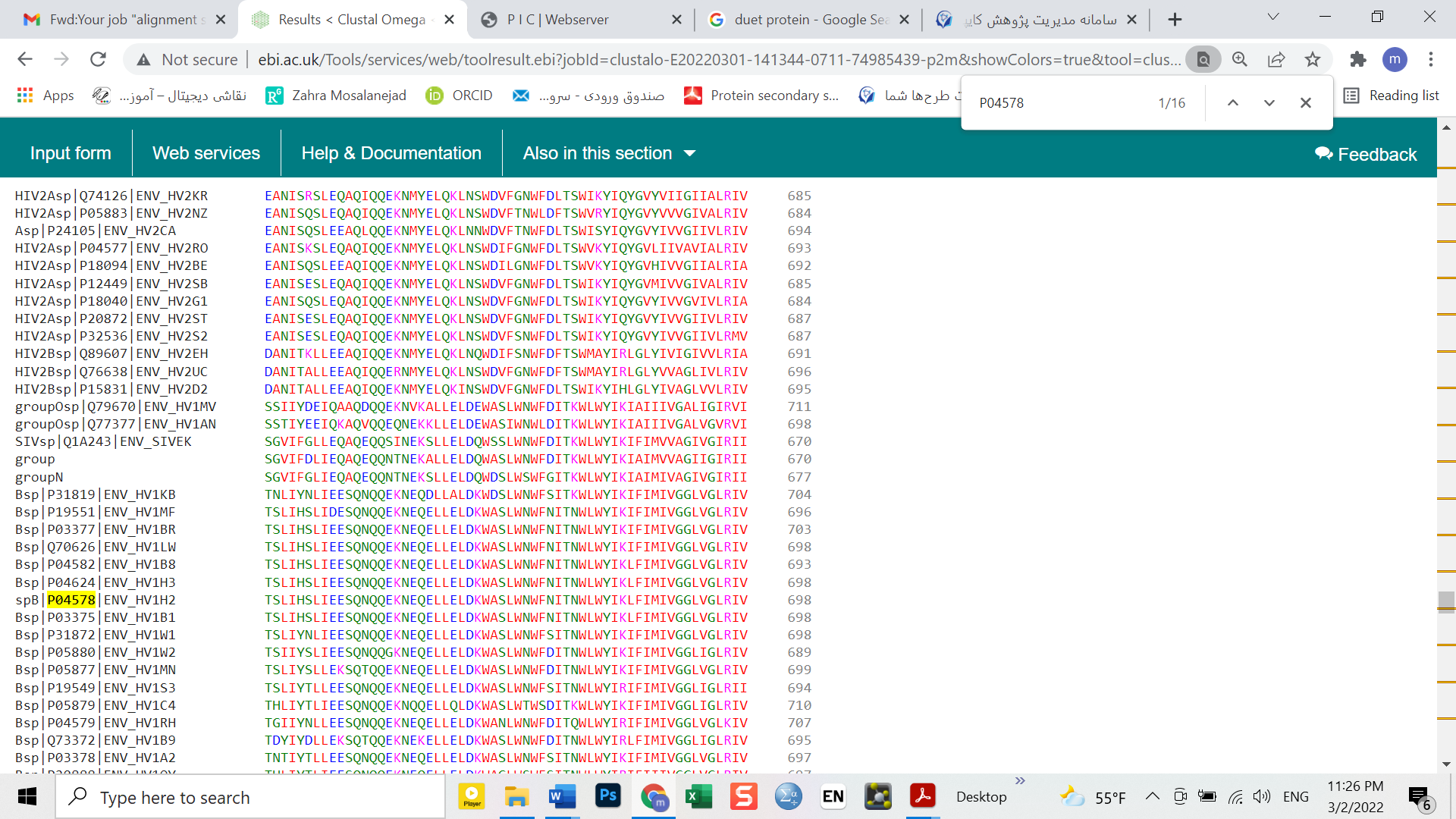


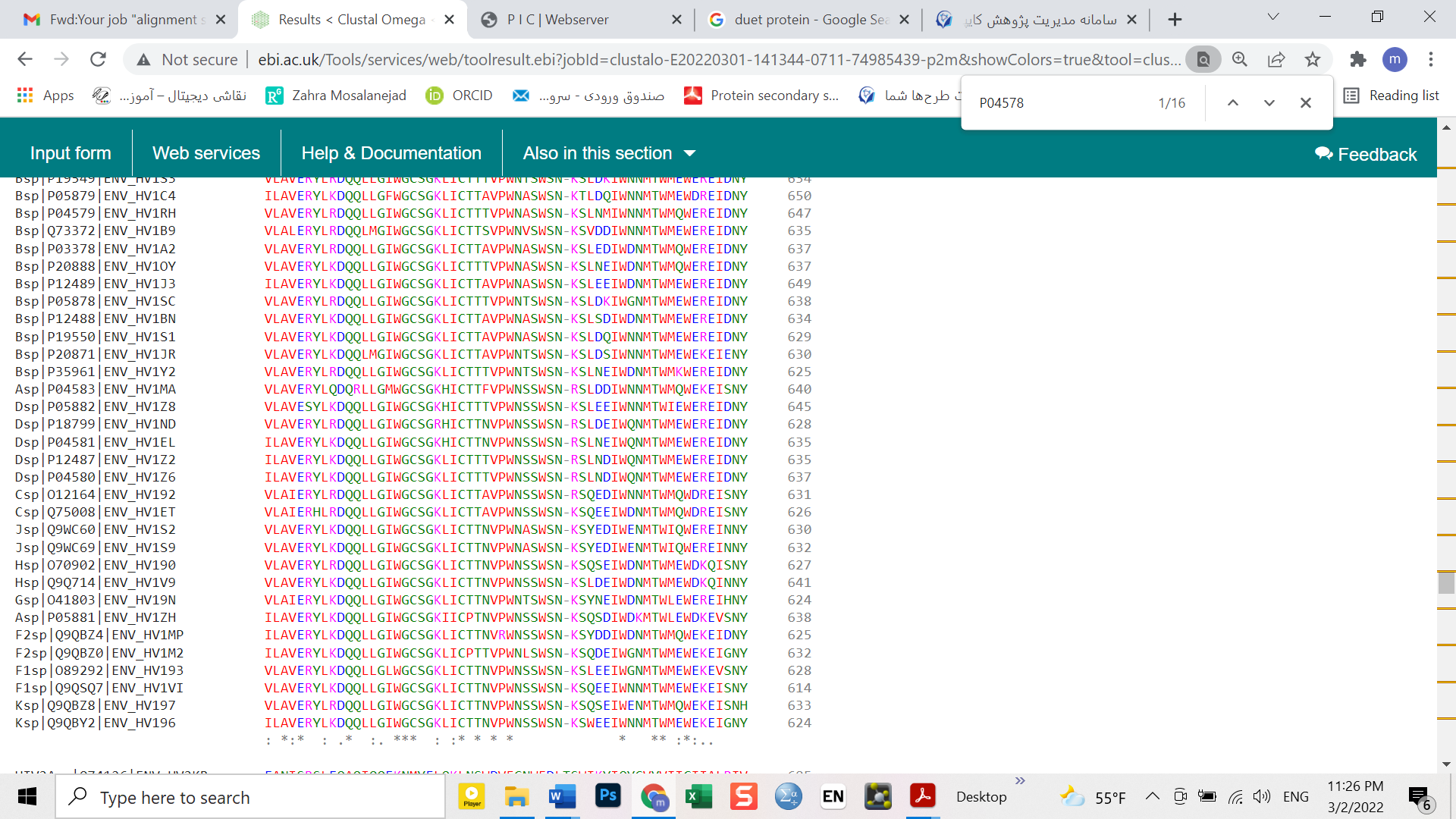

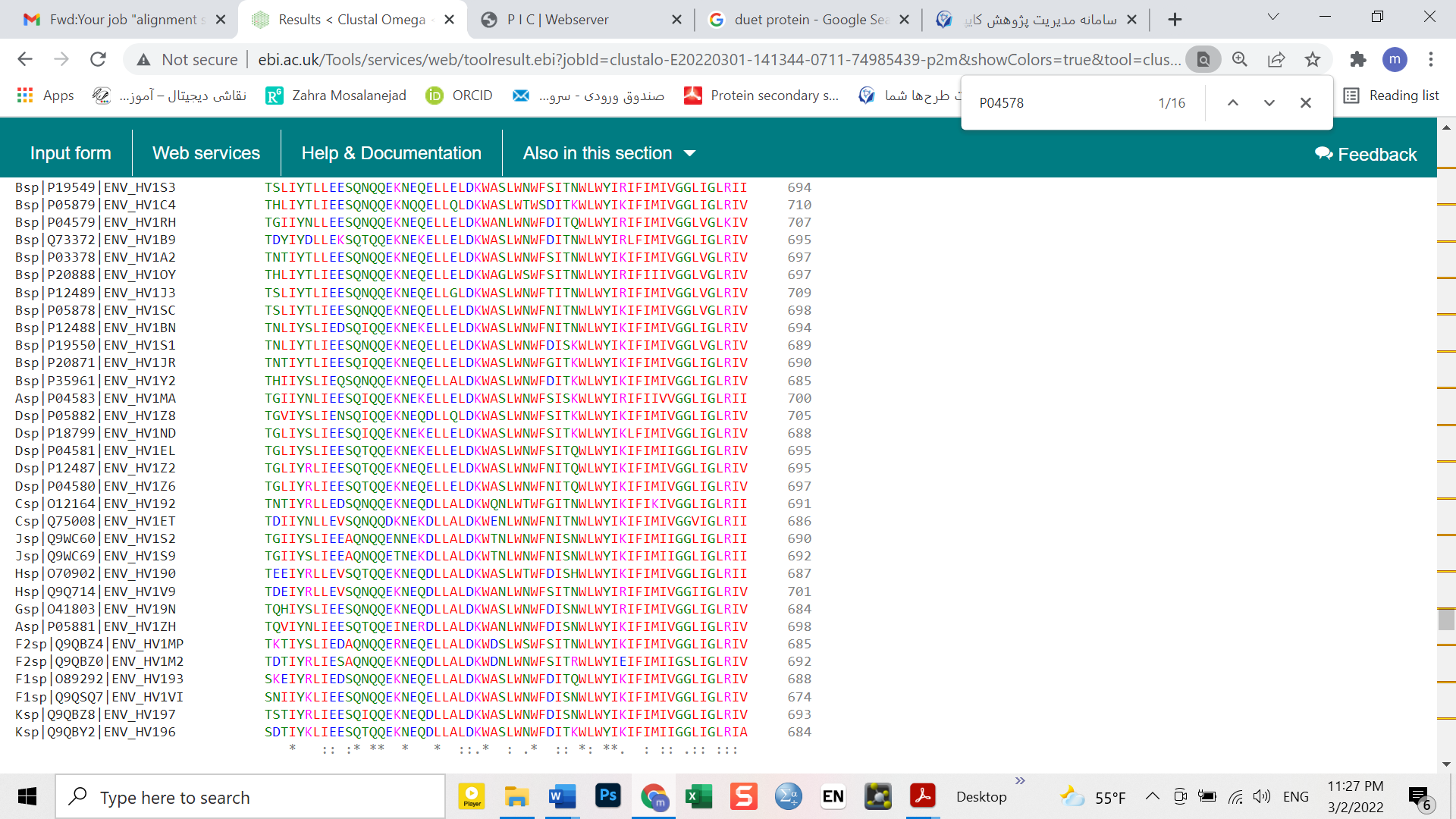


**Figure S4.** Alignment of different subtypes of Gp41, the first part, highlighted uniprot code p04578, corresponds to the sequence of GP41 used in this article. Numbers 628 to 661 are related to the CHR sequence used in docking.

| Each retrocyclin contains two contiguous segments: RCICGRGIC from the DEFT gene 1, 2, 3, 5, or 6 and/or RCICGRRIC from the DEFT-4 gene. Seven of these nine amino acids are conserved during evolution, and only the amino acid at position 5, (gly, arg, thr, val) and amino acid position 8, (ile, val or phe) are significantly different. These variable amino acids are in the turn part of the structure and conserved or more stable amino acids are placed in antiparallel beta strands. Counting should be done from the places that are arrowed in the figure below in the designated path (1).  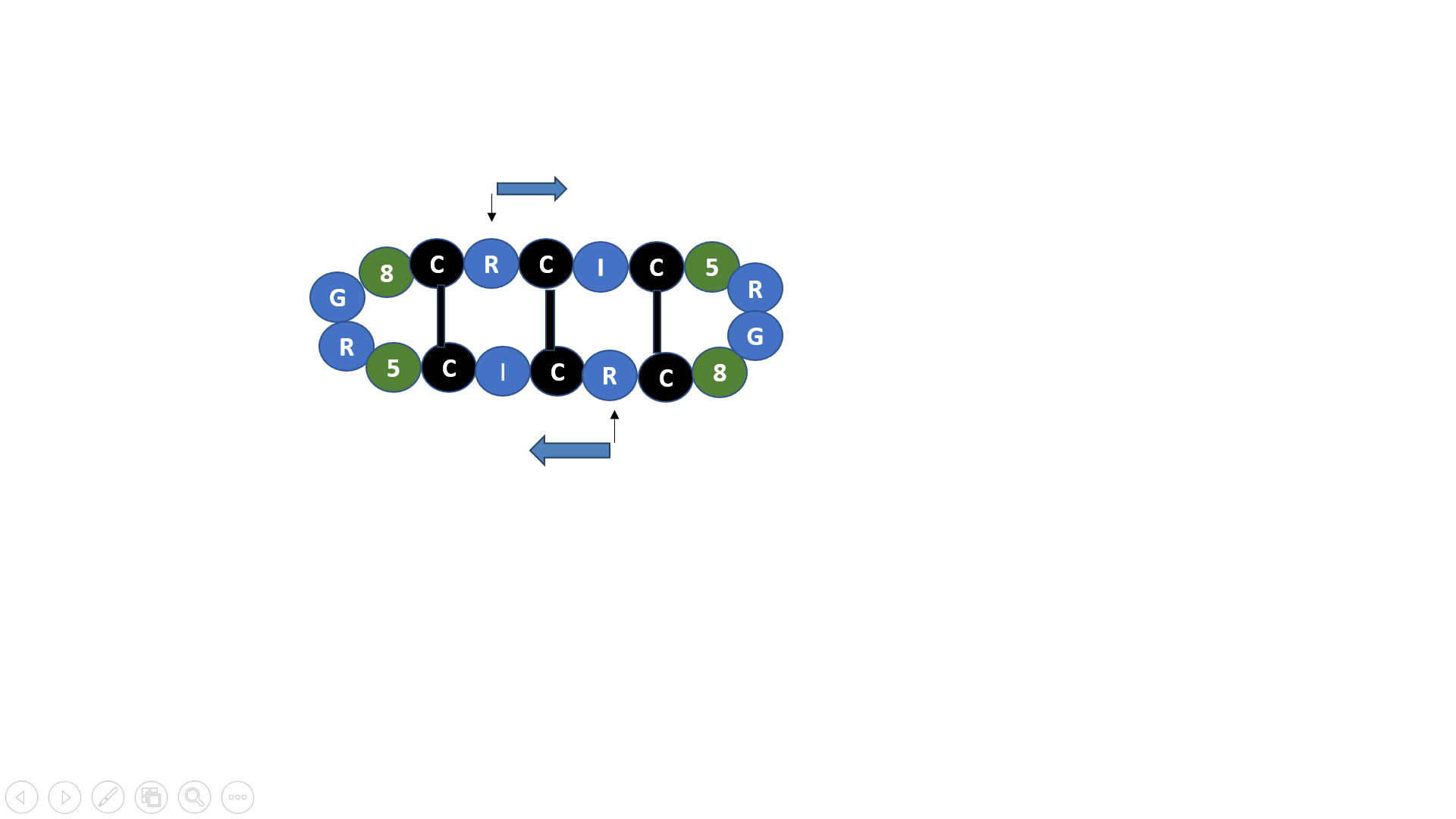 |
| --- |
| x-C-x-C-x (4)-C  Nona peptide motif is a 9 peptides motif whose amino acid CYS ​​is conserved during evolution (2). |
| The cyclic cysteine ​​ladder motif backbone is restrained by constrained cross-disulfide and hydrogen bonds and exposes the side chains to the solvent. There is also limited interaction between different side chains (3). |
| RC□C□RG□C  Nona peptide motif is a 9 peptides motif whose amino acid CYS ​​is conserved during evolution (4). |
| RCICRRGVC Black: conserved region  ^1 2 3 4 5 6 7 8 9^  Theta Defensins are cyclic peptides of 18 amino acids formed by the head-to-tail joining of two segments that can be homo-dimeric or heterodimeric to increase their diversity. Non-peptide motif of Theta Defensins: RC[IVLF]C[RTGVL][RL]G[VFI] C showing both a conserved CYS ​​pattern and a conserved hydrophilic/hydrophobic pattern. Positions 1, 5 and 6 are mainly cationic and hydrophilic arginine amino acids, while positions 3, 7 and 8 are mainly hydrophobic.  The side chains of ARG and the tri disulfide ladder are located on opposite sides of the cyclic backbone plane and thus form a polarized structure. Two types of theta-defensin homodimer, (RCICRRGVC)2 is under positive selection and (RCVCTRGVC)2 is not under positive selection (2). |

**Table S1.** Important amino acids of Theta Defensins.

| Sequence CHR | Refrence |
| --- | --- |
| XWMEWDREINNYTSLIHSLIEESQNQQEKNEQELL  main helper | (5) |
| XWMEWDREINNYTSLIHSLIEESQNQQEKNEQELL  Conserved and involving in binding pocket conserved | (6) |
| XWMEWDREINNYTSLIHSLIEESQNQQEKNEQELL | (7) |
| XWMEWDREINNYTSLIHSLIEESQNQQEKNEQELL | (8) |
| 4 articles 2 or 3 articles  X**W**ME**W**DREINNYTSLIHSLIEESQNQQEKNEQELL  628 631 635 638 642 645 649 652 656 659 | all  articles |
| X**W**ME**W**DREINNYTSL**I**HSLIEE**S**QN**Q**QEK**N**EQELL  Red: Highly conserved amino acids Yellow: Conserved amino acids Blue: Almost conserved regions | Alignment results |

**Table S2.** CHR’s essential amino acids in CHR NHR interaction.


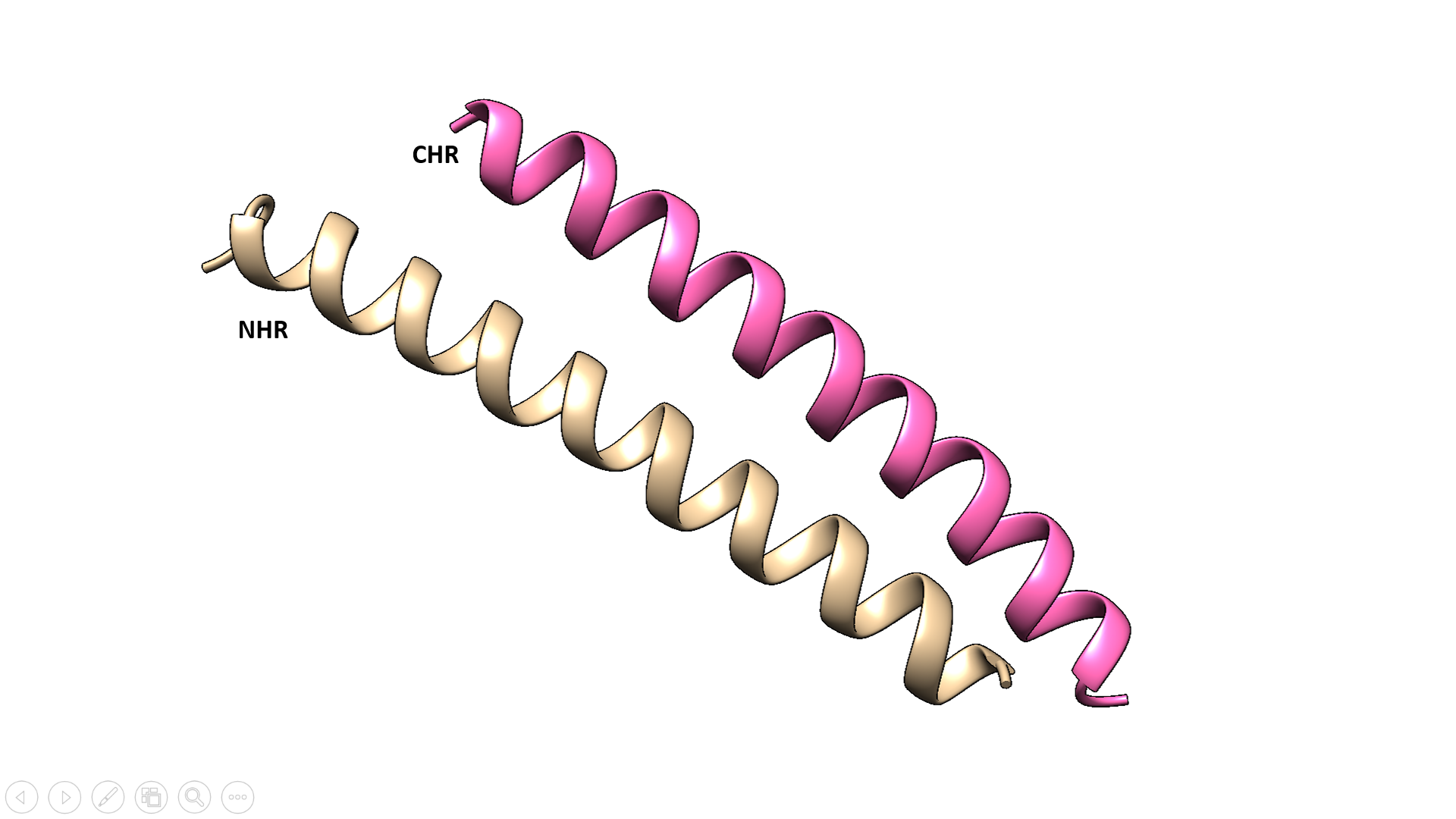


**Figure S5.** The structure of GP41 in the form of ribbons, glycoprotein 41 GP part (CHR part, pink color and cream color NHR)

| _aa server_ | 2 | 4 | 6 | 8 | 9 | 10 | 11 | 13 | 15 | 17 | 18 |
| --- | --- | --- | --- | --- | --- | --- | --- | --- | --- | --- | --- |
| Duet  ∆∆G>0 | R P N K L D E H Q V A | I V L T K P Q E Y N G A | R N | V A | L I V A | ----- | R N K V H D Q E | I L V E Q Y T N K M D | R N V Q K L | A V | L I V P N A R Q G |
| Cupsat | P W R | V W Y C | V W F Y C | V W F Y C | --- | ---- | C | V I Y C H | Y C | F N H | T F Y N R H |

**Table S3.** The amino acids proposed by DUET and CUPSAT servers are written in the table. ∆∆G>0 means more stability after mutation.

| 659 | 651 | 648 | 646 | 636 | 644 | 641 | 640 | Number |
| --- | --- | --- | --- | --- | --- | --- | --- | --- |
| GLU | ASN | GLU | ILE | ASN | SER | LEU | SER | Amino Acids |

**Table S4.** Amino acids susceptible to mutation in the CHR, taken from the Hot spot wizard data base.

| _Peptide_ | **_Grand average of hydropathicity_** **_GRAVY_** | **_Aliphatic index_** | **_Instability index_** | **_Estimated half-life (mammalian reticulocytes,_ *_in vitro_*_)_** | **_Molecular weight_**  **_g/mol_** | **_Theorical pI_** | **_Total number of positively charged amino acids (Arg + Lys)_** | **_Total number of negatively charged amino acids_**  **_(Asp + Glu)_** | _Toxicity_ | _Net charge = 7pH_ | _Estimated water solubility_ | _Allergenicity_ | _Antigenicity (Bacteria )_ | _Antigenicity (virus)_ | _Antigenicity (Tumor)_ | _Antigenicity_  _(Fungal)_ | _Antigenicity_  _)Parasite)_ |
| --- | --- | --- | --- | --- | --- | --- | --- | --- | --- | --- | --- | --- | --- | --- | --- | --- | --- |
| 2lzi | 0.517 | 86.67 | 36.55 stable | 30 | 2041.58 | 9.30 | 5 | 0 | non toxic | 4.6 | Good | --- | antigen | --- | --- | antigen | --- |
| RC101 | 0.778 | 86.67 | -9.42  stable | 30 | 1914.43 | 8.98 | 4 | 0 | non toxic | 3.6 | Good | allergen | antigen | --- | --- | antigen | --- |
| 0 | 0.250 | 43.33 | 6.87  stable | 30 | 2087.52 | 8.72 | 3 | 0 | toxic | 2.7 | Poor | allergen | --- | --- | --- | antigen | --- |
| 1 | -0.189 | 21.67 | 56.24  unstable | 30 | 2180.56 | 8.36 | 2 | 0 | toxic | 1.9 | Poor | --- | --- | --- | --- | antigen | Antigen |
| 2 | -0.211 | 21.67 | 43.09  unstable | 30 | 2157.53 | 8.35 | 2 | 0 | toxic | 1.9 | Poor | --- | --- | --- | --- | antigen | Antigen |
| 3 | -0.228 | 21.67 | 69.40  unstable | 30 | 2171.60 | 8.69 | 3 | 0 | toxic | 2.8 | Poor | allergen | --- | --- | --- | antigen | --- |
| 4 | -0.300 | 21.67 | 51.29  unstable | 30 | 2190.64 | 8.96 | 4 | 0 | toxic | 2.8 | Poor | --- | --- | --- | --- | antigen | --- |
| 5 | -0.072 | 21.67 | 33.42  stable | 30 | 2091.51 | 8.69 | 3 | 0 | toxic | 2.7 | Poor | allergen | --- | --- | --- | antigen | Antigen |
| A | 0.494 | 65.00 | 5.99  stable | 30 | 2056.55 | 9.01 | 4 | 0 | non toxic | 3.7 | Good | allergen | --- | --- | --- | antigen | --- |
| E | 0.489 | 65.00 | -4.71  stable | 30 | 2019.57 | 9.22 | 5 | 0 | non toxic | 4.6 | Good | allergen | --- | --- | --- | antigen | --- |
| H | 0.478 | 65.00 | -4.71  stable | 30 | 2033.51 | 9.01 | 4 | 0 | non toxic | 3.6 | Good | allergen | --- | --- | --- | antigen | --- |
| M | 0.550 | 65.00 | -4.71  stable | 30 | 2014.47 | 8.72 | 3 | 0 | non toxic | 2.7 | Poor | allergen | --- | --- | --- | antigen | --- |

**Table S5.** Investigating properties of designed peptide (toxicity by toxinpred, Examining the physicochemical properties by the Protparam, solubility and charge by pepcalc) Evaluating the possibility of allergenicity using AllerTOP server and predicting types of antigenicity with vaxijen.


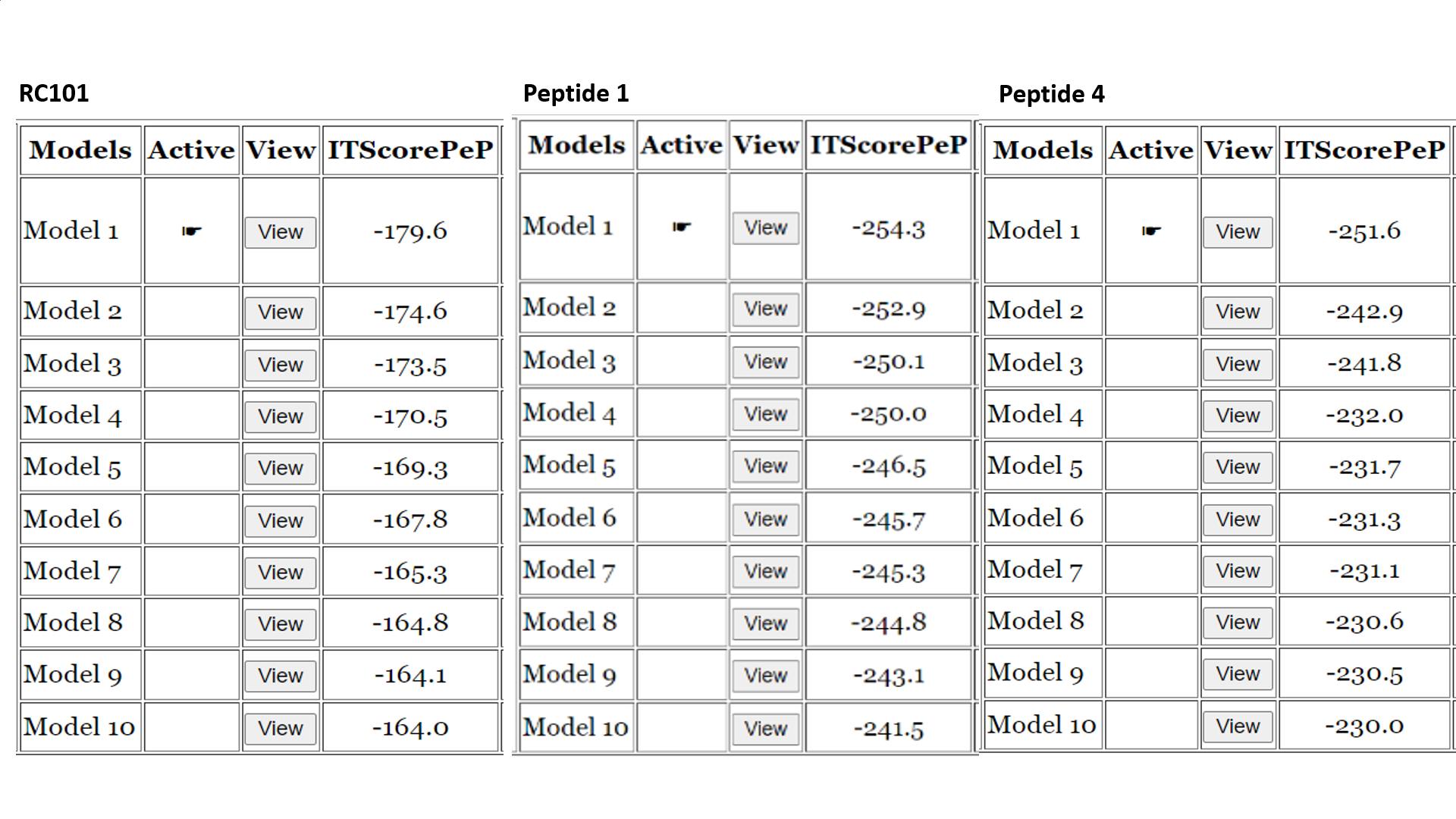


**Figure S6.** Top ten possible models of docking results of peptides (1 ,4 and RC101) with CHR in MDocKpep server. First model has the strongest binding affinity to CHR**.**

1. Nguyen TX, Cole AM, Lehrer RI. Evolution of primate θ-defensins: a serpentine path to a sweet tooth. Peptides. 2003;24(11):1647-54.

2. Cheng D-Q, Li Y, Huang J-F. Molecular evolution of the primate α-/θ-defensin multigene family. PLoS One. 2014;9(5):e97425.

3. Conibear AC, Rosengren KJ, Harvey PJ, Craik DJ. Structural characterization of the cyclic cystine ladder motif of θ-defensins. Biochemistry. 2012;51(48):9718-26.

4. Garcia AE, Osapay G, Tran PA, Yuan J, Selsted ME. Isolation, synthesis, and antimicrobial activities of naturally occurring θ-defensin isoforms from baboon leukocytes. Infection and immunity. 2008;76(12):5883-91.

5. Pu J, Wang Q, Xu W, Lu L, Jiang S. Development of protein-and peptide-based HIV entry inhibitors targeting gp120 or gp41. Viruses. 2019;11(8):705.

6. Holguín A, De Arellano ER, Soriano V. Amino acid conservation in the gp41 transmembrane protein and natural polymorphisms associated with enfuvirtide resistance across HIV-1 variants. AIDS research and human retroviruses. 2007;23(9):1067-74.

7. Liu S, Jing W, Cheung B, Lu H, Sun J, Yan X, et al. HIV gp41 C-terminal heptad repeat contains multifunctional domains: relation to mechanisms of action of anti-HIV peptides. Journal of Biological Chemistry. 2007;282(13):9612-20.

8. Su S, Wang Q, Xu W, Yu F, Hua C, Zhu Y, et al. A novel HIV-1 gp41 tripartite model for rational design of HIV-1 fusion inhibitors with improved antiviral activity. AIDS. 2017;31(7):885-94.
